# Home-monitoring of vital capacity in people with a motor neuron disease

**DOI:** 10.1007/s00415-022-10996-1

**Published:** 2022-02-07

**Authors:** Jochem Helleman, Jaap N. E. Bakers, Evelien Pirard, Leonard H. van den Berg, Johanna M. A. Visser-Meily, Anita Beelen

**Affiliations:** 1 Department of Rehabilitation, Physical Therapy Science and Sports, UMC Utrecht Brain Center, University Medical Center Utrecht, Heidelberglaan 100, 3584 CX Utrecht, The Netherlands; 2 Center of Excellence for Rehabilitation Medicine, UMC Utrecht Brain Center, University Medical Center Utrecht, and De Hoogstraat Rehabilitation, Utrecht, The Netherlands; 3 Revant Center for Rehabilitation, Breda, The Netherlands; 4 Department of Neurology, UMC Utrecht Brain Center, University Medical Center Utrecht, Utrecht, The Netherlands

**Keywords:** Motor neuron disease, Amyotrophic lateral sclerosis, Respiratory function, Vital capacity, Remote monitoring, Validity

## Abstract

**Background:**

Home-monitoring of spirometry has the potential to improve care for patients with a motor neuron disease (MND) by enabling early detection of respiratory dysfunction and reducing travel burden. Our aim was to evaluate the validity and feasibility of home-monitoring vital capacity (VC) in patients with MND.

**Methods:**

We included 33 patients with amyotrophic lateral sclerosis, progressive muscular atrophy or primary lateral sclerosis who completed a 12-week home-monitoring protocol, consisting of 4-weekly unsupervised home assessments of VC and a functional rating scale. At baseline, during a home visit, patients/caregivers were trained in performing a VC test, and the investigator performed a supervised VC test, which was repeated at final follow-up during a second home visit. Validity of the unsupervised VC tests was evaluated by the differences between supervised and unsupervised VC tests, and through Bland–Altman 95% limits-of-agreement. Feasibility was assessed by means of a survey of user-experiences.

**Results:**

The 95% limits-of-agreement were [− 14.3; 11.7] %predicted VC, and 88% of unsupervised VC tests fell within 10%predicted of supervised VC. 88% of patients experienced VC testing as easy and not burdensome, however, 15% patients did not think their VC test was performed as well as in the clinic. 94% of patients would like home-monitoring of VC in MND care.

**Discussion:**

Unsupervised VC testing at home, with prior face-to-face training, is a valid and time-efficient method for the remote monitoring of respiratory function, and well-accepted by patients with MND and their caregivers.

## Introduction

In patients with a motor neuron disease (MND), respiratory failure is the main cause of death [1, 2]. When patients show signs or symptoms of respiratory dysfunction, as described in clinical guidelines, non-invasive ventilation (NIV) is recommended [3–5]. Studies have shown that the use of NIV prolongs survival and improves quality of life [6–8]. Regular monitoring of respiratory function is essential to ensure timely detection of respiratory dysfunction so that NIV can be initiated [3–5]. In current MND healthcare, respiratory function is monitored during regular visits to a multidisciplinary clinic. Two drawbacks of this type of monitoring are that clinic visits can be time consuming and burdensome for patients with MND, and that patients have to visit the clinic irrespective of whether there is a decrease in respiratory function [9, 10]. This suggests that current respiratory monitoring may be insufficiently tailored to the needs of patients.

A potential solution is the home-monitoring of respiratory function through the use of telehealth. This approach allows for more frequent assessments, higher continuity of monitoring, especially when patients are not able to visit the clinic, and easy communication between patients and the multidisciplinary care team [11–16]. The use of telehealth may help to detect respiratory function decline early, and schedule clinic visits and initiate clinical interventions on time. One method of home-monitoring is the assessment of patient-reported symptoms of dyspnea, which was found to be useful for screening whether patients with MND had reduced vital capacity (VC) [17]. However, a drawback of dependence on self-reported dyspnea/orthopnea is that patients with low VC but without symptoms will not be identified (false negative rate = 14%). For this reason, combining patient-reported symptoms of dyspnea with home-based VC testing may reduce false negative findings and improve the home-monitoring of respiratory function.

The VC test has prognostic value in patients with MND [18, 19], and is easy to perform with a handheld spirometer, which is affordable and widely available. For these reasons, the VC test is suitable and relevant for home-monitoring; however, in MND care, its application for home-monitoring is still lacking [20]. Recently, a study showed that during COVID-19, it was feasible to perform home-based VC testing with supervision via video and that it was well-received by patients with MND in a healthcare setting [21]. However, one trial showed that when patients performed VC tests at home without supervision, the remote VC measurement was significantly higher than the usual in-clinic VC measurement and compliance was suboptimal [22]. These findings show the potential of home-monitoring of VC, but also indicate that more evidence is needed to support its implementation.

The aim of the present study is, therefore, to evaluate the validity and feasibility of unsupervised home-monitoring of VC in patients with MND.

## Methods

### Study design and population

This prospective cohort study aimed to include patients with MND, who were 18 years old or over and had access to a smartphone or tablet. Different diagnoses of MND were involved: amyotrophic lateral sclerosis (ALS), progressive muscular atrophy (PMA) and primary lateral sclerosis (PLS). The exclusion criteria were the use of non-invasive ventilation during the daytime, tracheostomy, or the inability to perform a VC test with or without caregiver assistance. Ethics approval from the Medical Ethics Committee of the University Medical Center Utrecht was obtained prior to the start of the study and patients gave their informed consent before participating.

### Setting and procedure

This study was conducted by the University Medical Center in Utrecht, the Netherlands, in collaboration with the Revant Center for Rehabilitation in Breda. Both centers have a multidisciplinary care team, coordinated by a physician. All study activities were performed at the patients’ homes, meaning that patients could participate in the present study without having to visit a multidisciplinary clinic. Patients who, between August 2020 and February 2021, received MND care from the multidisciplinary care teams were invited by the treating physician to participate. Most patients had access to the telehealth service *ALS Home-monitoring and Coaching* as part of their usual care. This telehealth service included the mobile ALS app for self-monitoring and messaging, which facilitated remote monitoring and communication between the patient and the multidisciplinary care team. A full description of *ALS Home-monitoring and Coaching* is available in a previous publication [12].

### Study assessments

**Fig. 1:**
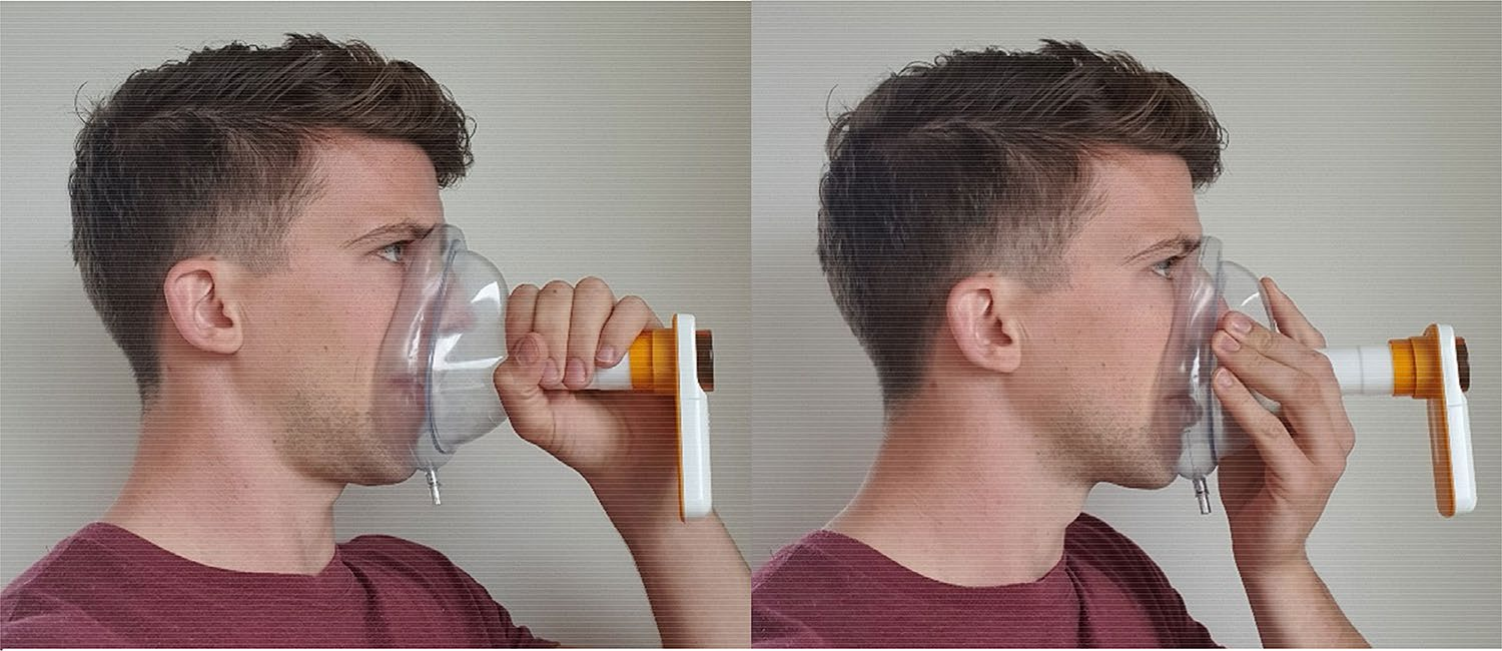
Performing a vital capacity test with a full-face mask. Left: A hammer grip around the tube. Right: Holding the mask with the tube placed between the fingers

Respiratory function was assessed by making three attempts to perform the vital capacity (VC) test in upright position, using a low-cost (ca. €100,-) handheld spirometer with Bluetooth connection to a mobile app (Spirobank Smart^®^, Medical International Research, Italy). The VC tests were performed with a full-face mask (Fig. 1) to enable testing in patients with bulbar impairment [23]. Patients recorded the time, date and VC test scores on a paper form, and also sent the VC test scores digitally to their multidisciplinary care team via the ALS app or by email, which allowed members of the multidisciplinary ALS care team to monitor respiratory function. The revised ALS functional rating scale (ALSFRS-R) was used to assess functional impairment [17, 24], and was self-monitored monthly as part of *ALS Home-monitoring and Coaching*. Patients who did not use telehealth completed the ALSFRS-R on paper at every follow-up. We created a survey to evaluate user-experiences of patients and caregivers who assisted with VC testing; see Tables 2 and 3 for the items of the survey. Items were scored on a 5-point Likert scale: the extent to which patients/caregivers considered aspects of VC testing to be difficult (answer options: Very easy–Very difficult), or the extent to which they agreed with a statement on VC testing (answer options: Totally agree–Totally disagree).

### Baseline protocol

At baseline (T0), the supervised VC test and ALSFRS-R were completed during a home visit. The investigator helped patients to install the mobile app on their smartphone, after which the supervised VC was performed. VC tests were either performed forcefully (FVC) or slowly (SVC), depending on which method was most effective/suitable for the patient [19]. Patients (and their caregivers) were instructed on how to perform the VC test independently, and practiced VC testing. If required, the investigator gave tips on how to improve the way the VC test was performed. When proper technique was observed (e.g. correct placement of mask, maximal in and exhalation, upright body position), the investigator left the room, and the patient performed an unsupervised VC test, to ensure that patients were able to do this without supervision. Unsupervised VC tests that were performed during the baseline home visit, were not included in the analysis.

### Follow-up protocol

The total follow-up period was 12 weeks, with 4-weekly unsupervised assessments. One day after the home visit (T1), patients completed their baseline unsupervised VC tests. At 4 weeks (T2), 8 weeks (T3) and 12 weeks (T4) after baseline, patients completed unsupervised VC tests and the ALSFRS-R. The investigator sent a reminder on the days of follow-up either via text-message or e-mail, depending on patient preference. At T4 the investigator visited the patient’s home at least 1 h after patients had completed their unsupervised VC tests. During this final home visit, a supervised VC test was performed and patients (and their caregivers) were asked to fill in the survey on user-experiences and to indicate the average duration of their VC testing sessions.

### Analyses

The highest VC test score, out of three attempts, at each time-point was converted to a percentage of the predicted (%predicted) VC, using height, age, and ethnicity (reference values used from Global Lung Function Initiative 2012) [25, 26]. We used the unsupervised test at T1 as baseline, since the unsupervised VC tests performed at T0 may have been affected by the prior supervised VC tests. Validity of unsupervised VC testing was assessed through the Bland–Altman 95% limits-of-agreement and Lin’s concordance correlation coefficient (CCC) between the supervised VC at T0 and the unsupervised VC at T1, and between the supervised and unsupervised VC at T4. Based on clinical experience, we considered a maximal difference of 10%predicted between supervised and unsupervised VC as an acceptable limit of agreement, since this will allow healthcare professionals to determine a trend of VC over time when the VC is monitored at 4-weekly intervals. Additionally, the coefficient of variation of supervised VC testing in patients with MND was already 6.3%predicted in a previous study [27]. A paired t-test was conducted to assess the change in supervised and unsupervised VC between T0 and T4, and whether there was a systematic difference between supervised and unsupervised VC. Furthermore, we evaluated whether the agreement between supervised and unsupervised VC was different after 12 weeks of home-monitoring compared to baseline. To obtain insight into the variation in unsupervised VC testing over time, we used linear regression to determine the average slope over the 12 week period for each individual patient, and we calculated the standard error (SE) which indicates to what extent the VC values deviate from the linear regression line. We then ranked patients from lowest to highest SE and created a subgroup for each quartile (25%) of patients. These subgroups were used to create 4 separate plots for the longitudinal unsupervised VC data of individual patients to facilitate interpretation of the data. Furthermore, in the Bland–Altman plots, the subgroups are indicated for each data point (i.e. patient), to indicate whether greater variability showed larger differences between unsupervised and supervised VC. An alpha of < 0.05 was considered to be statistically significant. Feasibility of unsupervised home-based VC testing was determined through the adherence to the 4-weekly VC protocol, time cost of VC testing and user-experiences. Unsupervised VC testing was considered feasible when ≥ 75% of all unsupervised VC tests had been carried out, and each testing session completed within 20 min. An item of the user-experience survey was considered feasible when ≥ 75% of patients answered ‘(totally) agree’ on positive statements, ‘(totally) disagree’ on negative statements, and ‘(very) easy’ on difficulty statements.

## Results

We included 33 patients with MND, with an average age of 60.5 years, 79% of whom were male. 76% were diagnosed with ALS, 15% with PMA and 9% with PLS, and 78.8% had spinal onset. At baseline, three patients were on nightly NIV, and one patient started with nightly NIV during the study period. Most patients (88%) used telehealth as part of their usual care. All baseline patient characteristics are presented in Table 1. Nine patients were assisted with VC testing by a partner (*N* = 4), family member (*N* = 3) or a home nurse (*N* = 2). The mean change over the 12-week period for the ALSFRS-R total score was − 2.1 points.

**Table 1:** Baseline patient characteristics

Characteristic	Patients (*N* = 33)
Gender (male), *n* (%)	26 (78.8)
Age (years), mean(SD)	60.5 (13.2)
Diagnosis, *n* (%)	
ALS	25 (75.8)
PMA	5 (15.2)
PLS	3 (9.1)
Site of onset, *n* (%)	
Bulbar	7 (21.2)
Spinal	26 (78.8)
Nightly NIV, *n* (%)	3 (12.1)
Gastrostomy, *n* (%)	2 (6.1)
Telehealth use, *n* (%)	29 (87.8)
Respiratory function (% of predicted VC), mean (SD)	78.4 (25.6)
Disease duration from first symptoms (months), median (IQR)	35.6 (17.2–52.2)
ALSFRS-R, mean (SD)	35.9 (7.3)
ALSFRS-R (respiratory domain), mean (SD)	11.0 (1.3)

*ALS* amyotrophic lateral sclerosis, *PMA* progressive muscular atrophy, *PLS* primary lateral sclerosis, *NIV* non-invasive ventilation, *VC* vital capacity, *SD* standard deviation, *IQR* interquartile range, *MND* motor neuron disease, *ALSFRS*-*R* revised ALS functional rating scale

### Validity of unsupervised VC testing

**Fig. 2:**
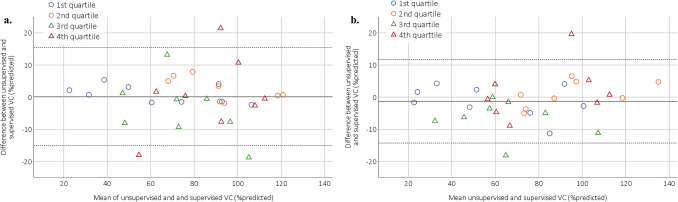
Bland–Altman plots. VC = vital capacity, Dashed line = 95% limits of agreement. The 4 quartile groups are based on the variability of the unsupervised VC scores over time, where 1st quartile = lowest variability and 4th quartile = highest variability. **a**. At baseline, **b**. at final follow-up

**Fig. 3:**
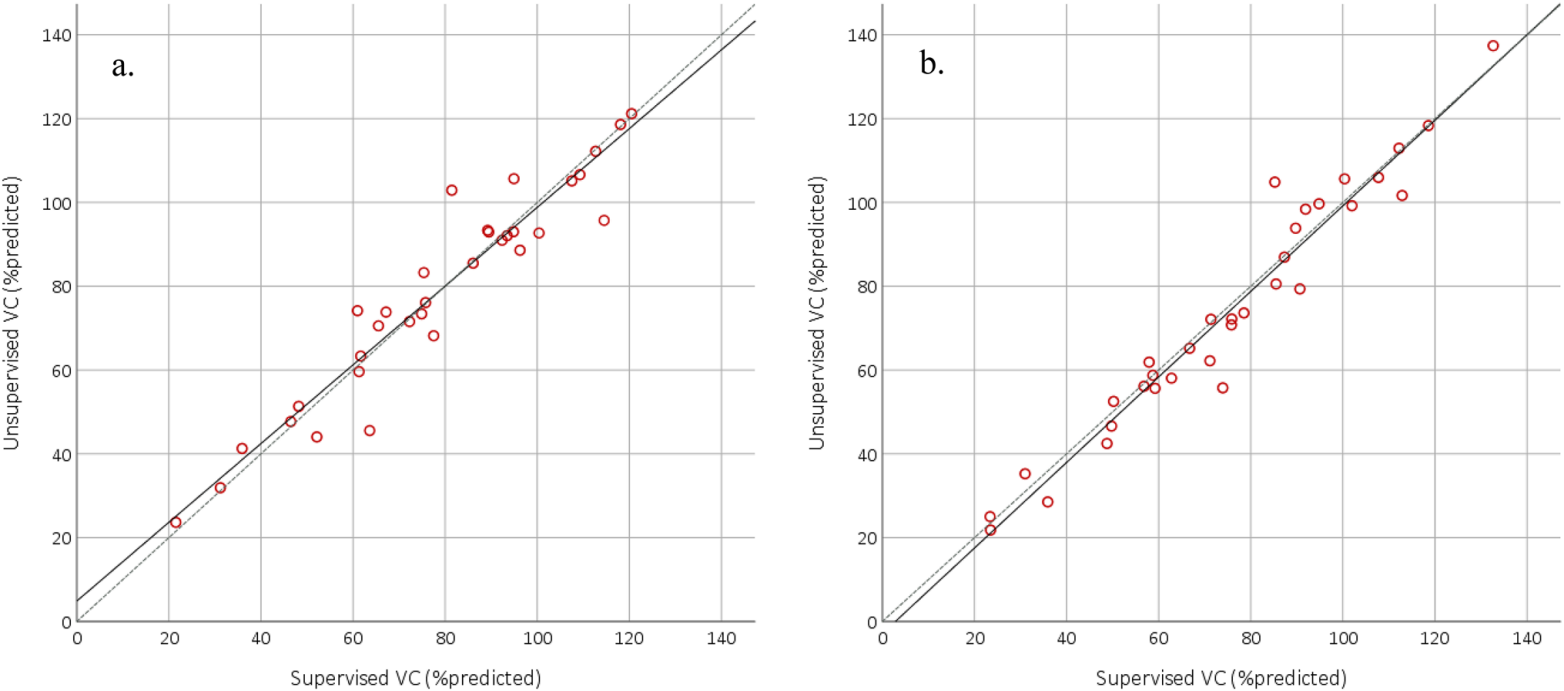
Scatterplot of unsupervised vs supervised vital capacity. *VC* vital capacity. Dashed line = line of identity. **a** At baseline, *Lin's CCC* = 0.953, **b** at final follow-up, *Lin's CCC* = 0.971

**Fig. 4:**
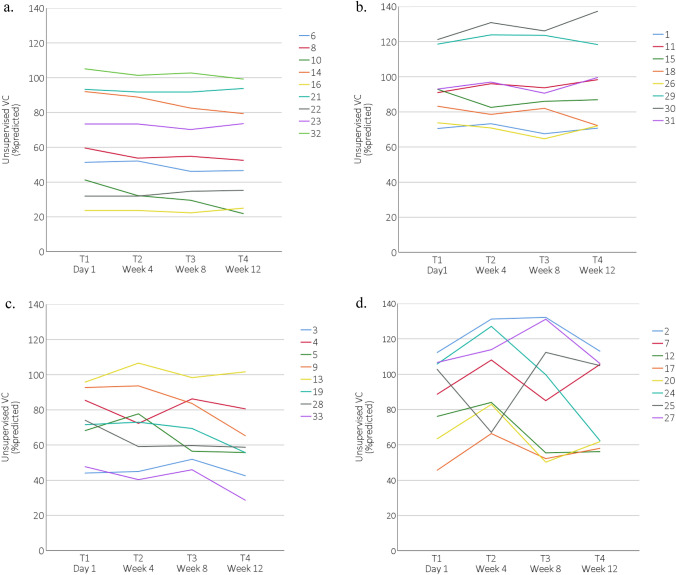
Unsupervised vital capacity over time per individual patient. VC = vital capacity. Patients were ranked from low to high variability, based on the standard error (SE) of the unsupervised VC scores over time and split into four quartiles (i.e. 25% of patients in each group). a) patients in the first quartile (SE range = 0.36–0.96 %predicted), b) patients in the second quartile (SE range = 1.02–2.16 %predicted), c) patients in the third quartile (SE range = 2.28–3.98 %predicted), and d) patients in the fourth quartile (SE range = 4.56–10.47 %predicted)

The 95% limits-of-agreement and the mean difference were [− 15.1; 15.4] and 0.12%predicted (*p* = 0.928) at baseline, respectively, and [− 14.3; 11.7] and –1.33%predicted (*p* = 0.259) at final follow-up, respectively (Fig. 2). The difference between unsupervised and supervised VC was smaller than 10%predicted in 28 of 33 (85%) patients at baseline and in 29 of 33 (88%) patients at final follow-up. The median absolute difference between supervised and unsupervised VC at baseline and final follow-up were 2.6 (IQR = 1.3–7.8) and 4.1 (IQR = 1.6–5.8) %predicted, respectively. Lin’s CCC was excellent at baseline (0.953), as well as at final follow-up (0.971) (Fig. 3). Between baseline and final follow-up both the supervised VC (Mean = − 3.31, *p* = 0.045) and unsupervised VC (-4.77, p = 0.036) decreased significantly. We also compared the change in supervised and unsupervised VC between baseline and final follow-up, which showed a good correlation (*ρ* = 0.74, *p* < 0.001). The plots of individual unsupervised VC data can be found in Fig. 4, where the range of SE was 0.36–0.96%predicted for the first quartile of patients, 1.02–2.16%predicted for the second quartile of patients, 2.28–3.98%predicted for the third quartile of patients, and 4.56–10.47%predicted for the fourth quartile of patients.

### Feasibility of home-monitoring

All 33 participants completed 100% of their VC assessments, 32 (97%) within 20 min, and 29 (88%) within 15 min. Patients reported that the spirometer and spirometry app were user-friendly, and that unsupervised VC testing was considered to be easy and not burdensome (Table 2). Most patients (30, 93.8%) would like their respiratory function from home for care purposes. Even patients with limited hand function were able to handle the spirometer and independently perform a VC test, as 29% (7/24) of patients who were not assisted by a caregiver had an ALSFRS-R fine motor score of ≤ 6. This was due to the fact that the face mask, which was attached to the mouthpiece of the spirometer, made it easier to hold the spirometer. Three patients experienced difficulties with determining whether a VC test was performed correctly and two patients felt insecure about their VC test performance in the absence of a healthcare professional. Furthermore, five patients did not think that the unsupervised VC tests were performed as well as supervised tests in the clinic.

**Table 2:** User-experiences of patients

Item	(Very) Easy *n* (%)	Neutral *n* (%)	(Very) Difficult *n* (%)	N*
Placing the mask on my face was	23 (82.1)	4 (14.3)	1 (3.6)	28
Handling the spirometer was	26 (92.8)	1 (3.6)	1 (3.6)	28
Starting a VC test in the app was	30 (96.8)	1 (3.2)	0 (0)	31
Performing a VC test was	29 (87.9)	3 (9.1)	1 (3)	33
Judging whether the test was performed correctly was	26 (78.8)	3 (9.7)	3 (9.7)	32

Item	(Totally) Agree *n* (%)	Neutral *n* (%)	(Totally) Disagree *n* (%)	N*
The spirometer is user-friendly	31 (93.9)	1 (3)	1 (3)	33
The spirometry app was user-friendly	30 (90.9)	3 (9.1)	0 (0)	33
The spirometer is appropriate for home-monitoring of respiratory function	30 (90.9)	3 (9.1)	0 (0)	33
I would like to monitor my respiratory function from home for care purposes	30 (93.8)	2 (6.3)	0 (0)	32
I know how to perform a VC test	33 (100)	0 (0)	0 (0)	33
I believe that my VC test at home is performed just as well as a usual VC test in the clinic	24 (72.8)	4 (12.1)	5 (15.1)	33
I am unsure about performing the VC test correctly in the absence of a healthcare professional	2 (6.5)	4 (12.9)	25 (80.6)	31
Performing VC tests at home is burdensome	2 (6.3)	2 (6.3)	28 (87.5)	32

*VC* vital capacity

*Missing data are due to patients answering “not applicable/ no opinion”

Based on the comments reported by patients during unsupervised VC testing, there were some difficulties that affected VC test performance: excessive mucus in throat (patient 4, Fig. 4c at T2), physical fatigue (patient 17, Fig. 4d at T1), pain in stomach caused by a gastrostomy tube (patient 24, Fig. 4d at T4), not being able to concentrate during testing (patient 26, Fig. 4b at T3), or physical discomfort due to an uncomfortable body position in wheelchair (patient 33, Fig. 4c at T4).

Most caregivers who assisted with VC testing reported that the spirometer (*n* = 7) and mobile app (*n* = 8) were user-friendly, and that helping with VC testing was easy (*n* = 7) and not burdensome (*n* = 7) (Table 3). The majority of caregivers believed they were able to (help) perform a VC test correctly (*n* = 8), and judge whether a VC test had been performed correctly (*n* = 7). Some of the caregivers (*n* = 3) did not think that they performed the unsupervised VC as well as a healthcare professional in a clinic.

**Table 3:** User-experiences of caregivers

Item	(Very) Easy	Neutral	(Very) Difficult
Placing the mask on his/her face was	8/9	1/9	1/9
Handling the spirometer was	8/9	0/9	1/9
Starting a VC test in the app was	8/8	0/8	0/8
Performing a VC test was	7/8	1/8	0/8
Judging whether the test was performed correctly was	7/9	2/9	0/9

Item	(Totally) Agree	Neutral	(Totally) Disagree
The spirometer is user-friendly	8/9	1/9	0/9
The spirometry app is user-friendly	7/8	1/8	0/8
The spirometer is appropriate for home-monitoring of respiratory function	8/9	1/9	0/9
I would like to monitor my respiratory function from home for care purposes	8/9	0/9	1/9
I know how to (help) perform a VC test	8/9	1/9	0/9
I believe that my VC test at home is performed just as well as a usual VC test in the clinic	5/9	1/9	3/9
I am unsure about performing the VC test correctly in the absence of a healthcare professional	1/9	1/9	7/9
Helping to perform VC tests at home is burdensome	0/9	2/9	7/9

Missing data are due to caregivers answering “not applicable/ no opinion”, *VC* vital capacity

## Discussion

The present study showed that unsupervised home-monitoring of VC, after one face-to-face training, was a valid method for the remote monitoring of respiratory function in patients with MND. Furthermore, the 4-weekly home-monitoring of VC without supervision was feasible, since adherence was excellent, and most patients and caregivers experienced VC testing as easy and not burdensome. Lastly, patients and caregivers were motivated to continue with home-monitoring of VC in MND healthcare.

Our results on the validity and feasibility of unsupervised VC testing at home are promising and show that this can be a time-efficient method in MND care for both patients and healthcare professionals for remotely monitoring respiratory function. We provided insight into the variation in unsupervised VC testing over time, which showed that most patients had a stable trend of VC during the 12-week period. However, the course of the unsupervised VC of some patients were highly variable over time, and generally showed larger differences with the supervised VC, indicating that these patients may require additional supervision during home-monitoring, e.g. through video.

We found that there was no systematic difference between unsupervised and supervised VC, but at final follow-up we observed that supervised VC test scores were more likely to be higher than the unsupervised VC test scores, when compared to baseline. This may indicate that the performance of the unsupervised VC test decreases over time in some patients. This finding is in contrast to previous studies, which reported that remote VC assessments were systematically higher than usual in-clinic VC assessments [22, 28]. An explanation for this finding, is that in the present study all VC tests were performed at patients’ homes, including the supervised tests. This limited the factors that may have negatively affected VC test performance, such as the burden of travelling and visiting a clinic.

We found that all patients adhered to the 4-weekly monitoring protocol, and that this frequency was acceptable. This corresponds to findings of a recent study, in which most patients reported that the highest acceptable frequency for remote respiratory assessments was monthly [21]. In the present study, facilitating factors for adherence to VC testing at home were that the spirometer and app were user-friendly, and VC testing was easy, not burdensome and not time consuming. A previous study reported suboptimal adherence with a weekly VC protocol, mainly due to connection problems and patients forgetting to complete measurements [22]. During our study we were fortunate that the spirometer and app only rarely malfunctioned, which resulted in re-doing a VC test, but never prevented patients from testing. Furthermore, the problem of forgetting a VC test was tackled by sending a reminder at each follow-up. Another facilitator for adherence was the fact that home-monitoring of VC was part of an existing telehealth service and that VC test results were monitored by the multidisciplinary care team. Patients are likely to be more motivated to complete assessments at home, when they know healthcare professionals are monitoring their data closely and will provide feedback when necessary [29].

During unsupervised home-monitoring there were several factors, unrelated to respiratory muscle weakness, which hindered optimal VC test performance, such as pain, physical fatigue or loss of concentration. This suggests that it is important that patients provide comments on their physical and psychological well-being at time of VC testing, to help healthcare professionals interpret VC scores remotely. Moreover, some patients and caregivers experienced difficulties with determining whether a VC test was performed correctly, and felt insecure about proper VC test performance without supervision. These patients may prefer access to online instruction-videos [30] or require video-supervision during home-monitoring, which has been shown to be well-accepted by patients with MND [21, 28]. A disadvantage of video-supervised monitoring, is that it takes healthcare professionals considerably more time, compared to unsupervised monitoring. Interestingly, one study reported that only a few patients felt they were able to perform a VC test at home without video-supervision, which contrasts with our study sample, where the majority believed they were able to perform a VC test at home without supervision. A reason for this discrepancy may be that patients in the present study were trained in unsupervised VC, and that most patients already had experience with telehealth and remote monitoring.

### Clinical implications

Our findings indicate that a single face-to-face training session prior to VC testing at home was sufficient for most patients to learn how to perform a VC test independently. In clinical practice, patients could be trained in VC testing during a visit to a multidisciplinary clinic or at home. Starting home-monitoring of VC shortly after diagnosis is most beneficial, as insight into the rate of disease progression can guide the timing of clinical interventions. When patients show noticeable or unexpected changes in their unsupervised VC during home-monitoring, a face-to-face or video consultation may be scheduled to determine whether a change in VC was caused by respiratory muscle weakness, or other factors, such as pain/discomfort, illness, fatigue or performing the VC test incorrectly. Support during VC testing at home could be improved by including MND-specific prompts, and written and visual feedback (e.g. flow-volume curve) in the mobile spirometry app.

Home-monitoring of VC could be combined with patient-reported symptoms of dyspnea, to provide healthcare professionals with more insight into the patient’s respiratory function and reduce false negative findings. When home-monitoring data indicates the presence of respiratory dysfunction, based on VC, symptoms or both, patients should be referred to a multidisciplinary clinic for further examination. An advantage of this approach is that the frequency and timing of clinic visits will be tailored to the rate of disease progression and needs of individual patients. In turn, this may result in earlier detection of a respiratory function decline, and more timely referral to a pulmonologist or initiation of NIV, compared to the usual 3 monthly in-clinic care. This study contributes to the recently published Road Map, which was created to facilitate the wide-scale adoption of digital technology and remote monitoring in MND, as it provides evidence on how to measure respiratory function in patients with MND [31].

### Strengths and limitations

A strength of the present study is that home-monitoring of VC was part of an existing telehealth service, which facilitated home-monitoring and communication, and optimized adherence. A limitation is the fact that the majority of patients in our cohort were male and relatively young, which reduces the generalizability of our results. Future studies could assess long-term home-monitoring of VC, and determine to what extent the course of unsupervised VC over time corresponds to disease progression, and how it relates to decision-making in MND care. We assessed the upright VC in the present study, despite studies showing that in some patients the upright FVC may remain stable even when respiratory insufficiency is already present [32–35]. Based on existing literature, the maximal inspiratory pressure (MIP), sniff nasal inspiratory pressure (SNIP) or supine VC may be more sensitive in detecting respiratory muscle weakness [9, 18, 27–29]. However, due to the lack of low-cost respiratory pressure meters, home-monitoring of MIP and SNIP will be much more costly. Furthermore, the supine VC test can be challenging and burdensome to perform for patients with gross motor disability, as it requires transfer to a flat surface. As a result, more patients may require assistance from a caregiver, which increases caregiver burden and may reduce adherence. However, future studies could evaluate whether other pulmonary function tests, besides the upright VC, are valid and feasible for home-monitoring in patients with MND.

## Conclusion

Unsupervised VC testing at home, with prior face-to-face training and reminders during follow-up, is a valid and feasible method for the remote monitoring of respiratory function in MND care, and well-received by patients and their caregivers.

## Data Availability

Not applicable.
